# Paracrine Signals From Liver Sinusoidal Endothelium Regulate Hepatitis C Virus Replication

**DOI:** 10.1002/hep.26571

**Published:** 2013-12-18

**Authors:** Ian A Rowe, Sukhdeep K Galsinh, Garrick K Wilson, Richard Parker, Sarah Durant, Catalin Lazar, Norica Branza-Nichita, Roy Bicknell, David H Adams, Peter Balfe, Jane A McKeating

**Affiliations:** 1 Hepatitis C Virus Research Group, Institute for Biomedical Research, University of BirminghamBirmingham, UK; 2 Centre for Liver Research and NIHR Birmingham Liver Biomedical Research Unit, Institute for Biomedical Research, University of BirminghamBirmingham, UK; 3 Angiogenesis Group, Institute for Biomedical Research, University of BirminghamBirmingham, UK; 4 Viral Glycoproteins Department, Institute of BiochemistryBucharest, Romania

## Abstract

Hepatitis C virus (HCV) is a major cause of global morbidity, causing chronic liver injury that can progress to cirrhosis and hepatocellular carcinoma. The liver is a large and complex organ containing multiple cell types, including hepatocytes, sinusoidal endothelial cells (LSEC), Kupffer cells, and biliary epithelial cells. Hepatocytes are the major reservoir supporting HCV replication; however, the role of nonparenchymal cells in the viral lifecycle remains largely unexplored. LSEC secrete factors that promote HCV infection and transcript analysis identified bone morphogenetic protein 4 (BMP4) as a candidate endothelial-expressed proviral molecule. Recombinant BMP4 increased HCV replication and neutralization of BMP4 abrogated the proviral activity of LSEC-conditioned media. Importantly, BMP4 expression was negatively regulated by vascular endothelial growth factor A (VEGF-A) by way of a VEGF receptor-2 (VEGFR-2) primed activation of p38 MAPK. Consistent with our *in vitro* observations, we demonstrate that in normal liver VEGFR-2 is activated and BMP4 expression is suppressed. In contrast, in chronic liver disease including HCV infection where there is marked endothelial cell proliferation, we observed reduced endothelial cell VEGFR-2 activation and a concomitant increase in BMP4 expression. *Conclusion*: These studies identify a role for LSEC and BMP4 in HCV infection and highlight BMP4 as a new therapeutic target for treating individuals with liver disease.

Hepatitis C virus (HCV) is a major cause of global morbidity and mortality, with an estimated 170 million infected individuals worldwide. HCV causes chronic liver injury that can progress to cirrhosis and is one of the leading causes of hepatocellular carcinoma (HCC).^1^,^2^ Multiple drugs targeting HCV replicase enzymes are currently in development and the outlook for many is favorable; however, combination therapies involving agents that target host cell pathways may limit the development of viral resistance and maximize treatment responses. Hence, there is a need to define host cell pathways used by HCV to aid the discovery of new targets for future intervention strategies.

The liver is a large and complex organ containing multiple cell types, including sinusoidal endothelial cells (LSEC), stellate cells, Kupffer cells, and biliary epithelial cells as well as hepatocytes. Hepatocytes are the major reservoir supporting HCV replication and the contribution of other cell types in the viral lifecycle remain largely unexplored.^3^ LSEC hepatocyte interactions are critical for normal liver development and function and regulate the organ's response to injury.^4^ One of the best-characterized paracrine signals between these endothelial and epithelial cells is vascular endothelial growth factor-A (VEGF-A), which maintains the sinusoidal endothelial fenestrated phenotype and promotes hepatocyte growth factor expression in response to injury.^4^–^6^

VEGF-A is a multifunctional cytokine originally described for its ability to increase endothelial permeability, and subsequently reported to have critical roles in vascular development and cancer angiogenesis.^7^ We previously reported that HCV infection promotes VEGF-A expression, resulting in hepatocyte depolarization and enhanced viral entry.^8^–^10^ Treating HCV-infected hepatocytes with VEGF-A inhibitors, including sorafenib, restored their ability to polarize and limited viral infection,^8^,^11^ suggesting a therapeutic role for VEGF-A inhibitors in HCV infection.

These reports led us to investigate the role of LSEC in the HCV lifecycle and to establish coculture systems to study endothelial-epithelial cell interactions. We discovered a new paracrine network that regulates HCV replication. LSEC express bone morphogenetic protein 4 (BMP4) that increases hepatocyte permissivity to support HCV replication. BMP4 is negatively regulated at the transcriptional level by VEGF-A activation of VEGF receptor-2 (VEGFR-2) and downstream p38 MAPK signaling. *Ex vivo* studies demonstrate increased BMP4 expression and reduced endothelial cell VEGFR-2 activation in the diseased liver, highlighting new aspects of LSEC-hepatocyte crosstalk that may limit the efficacy of anti-VEGF therapies in HCV infection and suggesting therapeutic manipulation of BMP4.

## Materials and Methods

### Clinical Material

Tissue for cell isolation or *ex vivo* analysis was obtained from patients undergoing liver transplantation for endstage liver disease, or from donor liver surplus to surgical requirements at the Queen Elizabeth Hospital, UK. Informed consent and regional Ethics Committee approvals were given.

### Cell Culture

LSEC were isolated from donor liver tissue by enzymatic digestion, density centrifugation, and immunomagnetic separation.^12^ Purity was greater than 95% as judged by expression of the LSEC specific lectin L-SIGN. Cells were routinely cultured in human endothelial basal media (Invitrogen) supplemented with 10% human serum, VEGF-A, and hepatocyte growth factor (HGF) (both 10 ng/mL, Peprotech) on tissue culture plastic coated with rat tail collagen (Sigma), unless otherwise stated. Huh-7.5 cells (provided by Charles Rice, Rockefeller University) were propagated in Dulbecco's Modified Eagle Medium (DMEM) supplemented with 10% fetal bovine serum (FBS)/1% nonessential amino acids. Primary human hepatocytes were isolated using previously published protocols and maintained in Williams E medium supplemented with 10% FBS / 5 mM HEPES/insulin/dexamethasone. All cells were maintained at 37°C in 5% CO_2_. Cocultures were established by seeding cells at 4 × 10^4^/cm^2^ at a 1:1 ratio in human endothelial basal media supplemented with 10% human serum.

### Growth Factor and Pharmacologic Treatments

LSEC or Huh-7.5 cells were seeded at 4 × 10^4^/cm^2^ and allowed to adhere overnight in the absence of VEGF-A and HGF. The following day cells were incubated with growth factors: VEGF-A, placental growth factor (PlGF), bone morphogenetic protein-4 (BMP4) (all Peprotech), and VEGF-E (RELIATech) at 10 ng/mL unless otherwise stated. Following stimulation with growth factors or conditioned media, cells were treated with neutralizing antibodies targeting VEGF-A or BMP4 (R&D Systems) (10 μg/mL) as indicated. VEGF receptor (VEGFR) −1 (18F1) and VEGFR-2 (1121-B) neutralizing antibodies (ImClone Systems) were used as described.^13^,^14^ LSEC were treated with kinase inhibitors for 1 hour, the inhibitor removed, and cells stimulated with VEGF-A as indicated. Specifically these inhibitors target MEK1 (PD98059), p38 MAPK (SB203580), phospholipase C (PLC, U73122), and PI3 kinase (wortmannin). For the collection of conditioned media, cells were treated for 24 hours before harvest and stored at −20°C. Mock media was human endothelial basal media supplemented with 10% human serum that was incubated at 37°C for 24 hours. Conditioned media were diluted 1:2 with fresh media prior to use.

### Quantitative Reverse-Transcription Polymerase Chain Reaction (RT-PCR)

Purified RNA samples were amplified for target genes as indicated with commercial quantification kits (ABI), or HCV RNA (Primer Design) in a single tube RT-PCR in accordance with the manufacturer's instructions (Cells Direct kit, Invitrogen). Fluorescence was monitored in an MxPro-3000 PCR machine (Stratagene). Glyceraldehyde-3-phosphate dehydrogenase (GAPDH) was included as an endogenous control for amplification efficiency and RNA quantification.

### HCV Genesis and Infection

JFH-1 was generated as described.^15^ Briefly, RNA was transcribed *in vitro* from full-length genomes using the RiboExpress T7 kit (Promega) and electroporated into Huh-7.5 cells. Then 72 and 96 hours after electroporation supernatants were collected and stored immediately at −80°C. Virus-containing media were incubated with target cells at a multiplicity of infection (MOI) of ∼0.01. Infected cells were detected by methanol fixation and staining for viral NS5A with monoclonal antibody 9E10 (provided by Charles Rice, Rockefeller University) and Alexa-488 antimouse IgG. Infection was quantified by enumerating NS5A+ foci and infectivity defined as the number of focus-forming units/mL.

### Microarray Gene Expression Profile

An Agilent 44k genome microarray was used to analyze gene expression. LSEC and HUVEC, each from two independent donors, were seeded and treated with VEGF-A (10 ng/mL) for 18 hours. RNA was extracted using RNeasy Mini Kit (Qiagen) and labeled according to the manufacturer's instructions. RNA was hybridized to the array in accordance with the manufacturer's recommendations (Two-color Quick Amp labeling v. 5.7, Agilent). Transcripts regulated >2-fold and with a false-discovery rate of <10% were selected and analyzed using DAVID bioinformatics resources.^16^ Microarray data were uploaded to NCBI GEO (GSE41110 http://www.ncbi.nlm.nih.gov/geo).

### Immunohistochemistry

Formalin-fixed paraffin-embedded samples were obtained from liver tissue. Immunohistochemistry was performed as reported.^17^

### Immunoblotting

LSEC were harvested in CellLytic MT buffer (Sigma) containing protease and phosphatase inhibitors (Roche). Tissue samples were homogenized (GentleMACS, Miltenyi) in CellLytic MT buffer and lysates cleared by centrifugation. Proteins were separated by sodium dodecyl sulfate-polyacrylamide gel electrophoresis (SDS-PAGE), transferred to polyvinylidene membranes, probed with anti-VEGF, anti-BMP4 (R&D systems), anti-VEGFR-2, anti-Y1175 VEGFR-2, anti-VE-cadherin, anti-phospho p38 (all Cell Signaling), or anti-CD31 (Dako) as indicated and horseradish peroxidase (HRP)-conjugated secondary antibody. Proteins were detected by enhanced chemiluminescence (Geneflow).

### Statistics

Results are presented as mean ±1 SD. The Mann-Whitney *U* test or the Kruskal-Wallis test with Dunn's correction as appropriate were used for statistical analysis.

## Results

### LSEC Stimulate HCV Replication

**Figure 1:**
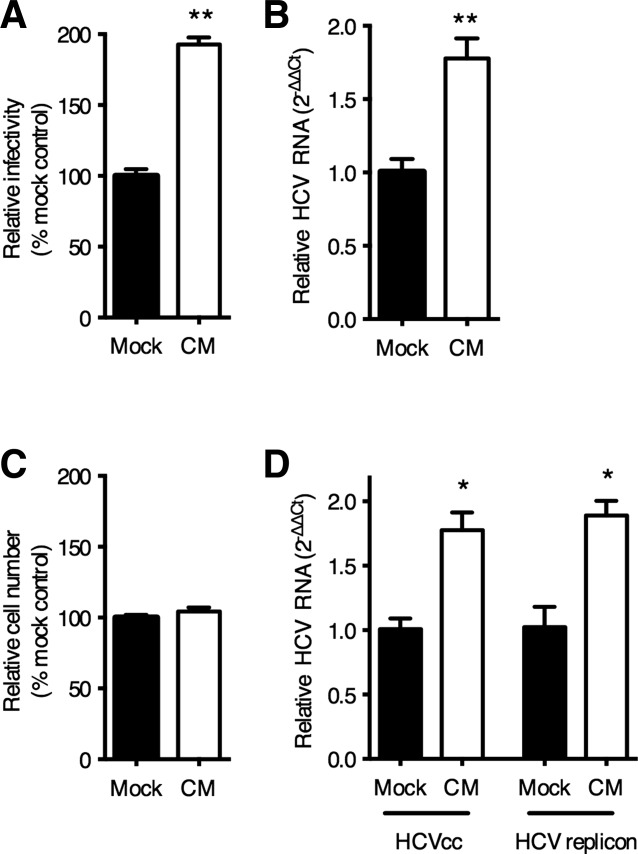
LSEC secreted factors increase hepatocellular HCV replication. Conditioned media (CM) was collected from LSEC seeded at 4 × 10^4^/cm^2^ for 24 hours in the absence of VEGF-A and was diluted 1:2 with fresh media and used to treat Huh-7.5 (A), or PHH (B) for 18 hours prior to infecting with HCV JFH-1. CM was replenished following infection. HCV infection was enumerated by quantifying NS5A expressing cells or HCV RNA levels as indicated and the data expressed relative to mock-treated cells. In replicate experiments with Huh-7.5, the number of cells following mock or CM treatment was quantified after 48 hours (C). Huh-7.5 supporting a JFH-1 subgenomic replicon were treated with CM for 48 hours and HCV RNA quantified (D). Data are mean ±1 SD of n = 4 donor LSEC CM. Statistical comparisons were made with the Mann-Whitney *U* test where *P < 0.05, and **P < 0.01 versus mock CM.

To investigate whether LSEC secrete factors that regulate HCV replication, Huh-7.5 hepatoma cells were incubated with conditioned media (CM) from LSEC, propagated in the absence of exogenous growth factor supplements, prior to infecting with HCV strain JFH-1. CM significantly increased HCV infection of both Huh-7.5 cells (Fig. 1A) and primary human hepatocytes (Fig. 1B) with no detectable effect on cell viability (Fig. 1C). Similar effects were observed with LSEC isolated from multiple donors (Supporting Fig. 1) and with human umbilical vein endothelial cells (data not shown), demonstrating that this effect is not specific to liver sinusoidal endothelial cells. CM increased the levels of HCV RNA in subgenomic replicon-bearing cells, suggesting that these factor(s) promote infection at the level of genome replication (Fig. 1D). To determine whether LSEC were able to stimulate replication of other hepatotropic viruses we tested LSEC CM in hepatitis B virus culture systems and failed to observe any modulation of replication (data not shown). These observations suggest a role for LSEC-derived soluble mediators in specifically regulating HCV replication.

### VEGF-A Limits LSEC Expression of Proviral Mediators

**Figure 2:**
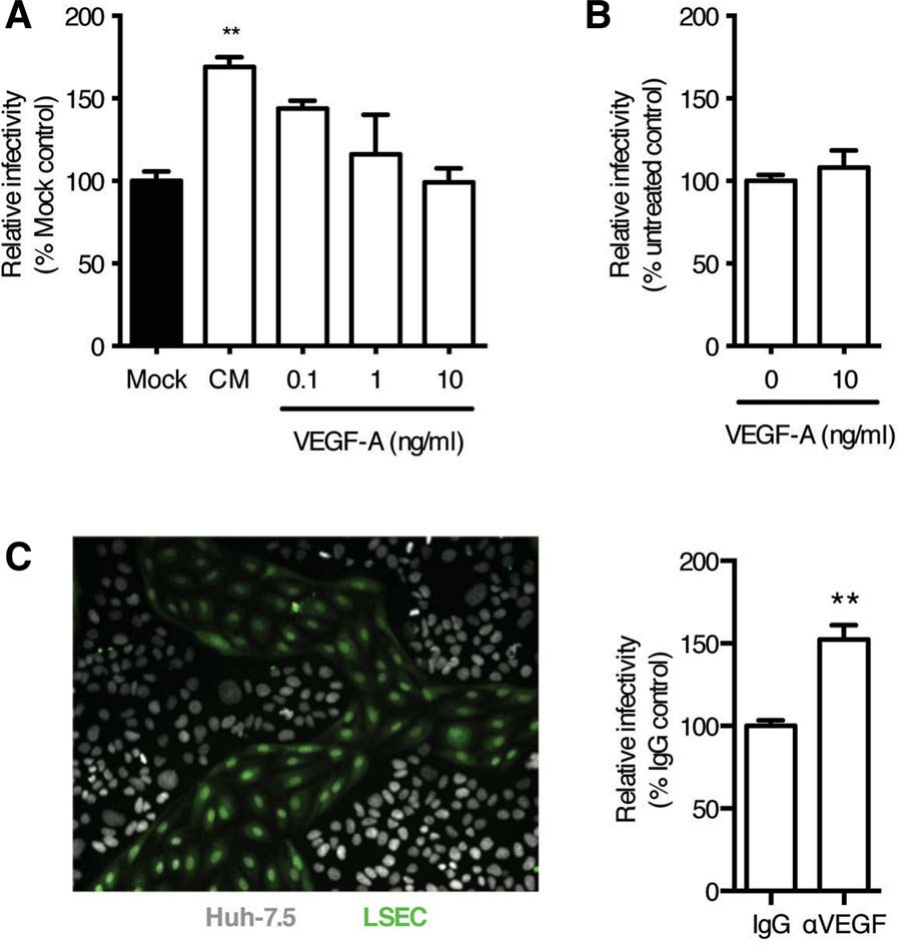
Paracrine VEGF signaling reduces HCV infection of LSEC-hepatocyte co-cultures. CM was collected from LSEC-treated with increasing concentrations of recombinant VEGF-A and screened for its effect on Huh-7.5 permissivity to support HCV JFH-1 infection. Infection was enumerated by quantifying NS5A-expressing cells and the data expressed relative to mock control (black) (A). Data are mean ±1 SD of n = 4 donor LSEC CM. Statistical comparisons were made with the Kruskal-Wallis test with Dunn's correction where **P < 0.01 versus mock CM. Recombinant VEGF-A (10 ng/mL) was used to treat Huh-7.5 cells at the time of HCV infection. Infectivity was assessed as above and expressed relative to untreated control (B). LSEC/Huh-7.5 cocultures were established and a representative image is shown where LSEC are labeled with CMFDA cell tracker dye (C). LSEC/Huh-7.5 cocultures were treated with neutralizing anti-VEGF antibody or irrelevant IgG (10 μg/mL) prior to infecting with HCV (C). Data are mean ±1 SD of n = 4 donor LSEC cocultures. Statistical comparisons with the Mann-Whitney *U* test where **P < 0.01 versus IgG control.

*In vivo* LSEC are continually exposed to hepatocyte-derived VEGF-A and this growth factor has been proposed to protect the liver against viral infection.^5^ We therefore measured the effect of VEGF-A on LSEC expression of proviral soluble factors. VEGF-A decreased the proviral activity of CM in a dose-dependent manner (Fig. 2A). In contrast, treating Huh-7.5 hepatoma cells with VEGF-A had no effect on HCV infection (Fig. 2B). To further study these paracrine signals, hepatoma cells were cocultured with LSEC (Fig. 2C) and evaluated for their ability to support HCV infection in the presence or absence of a neutralizing anti-VEGF-A antibody. We confirmed that LSEC do not support HCV infection (data not shown) and neutralization of VEGF-A increased HCV infectivity in the coculture system. These data are consistent with a down-regulation of endothelial expressed proviral factor(s) in the presence of VEGF-A signaling in LSEC.

### BMP4 Promotes HCV Replication and Is Suppressed by VEGF

**Figure 3:**
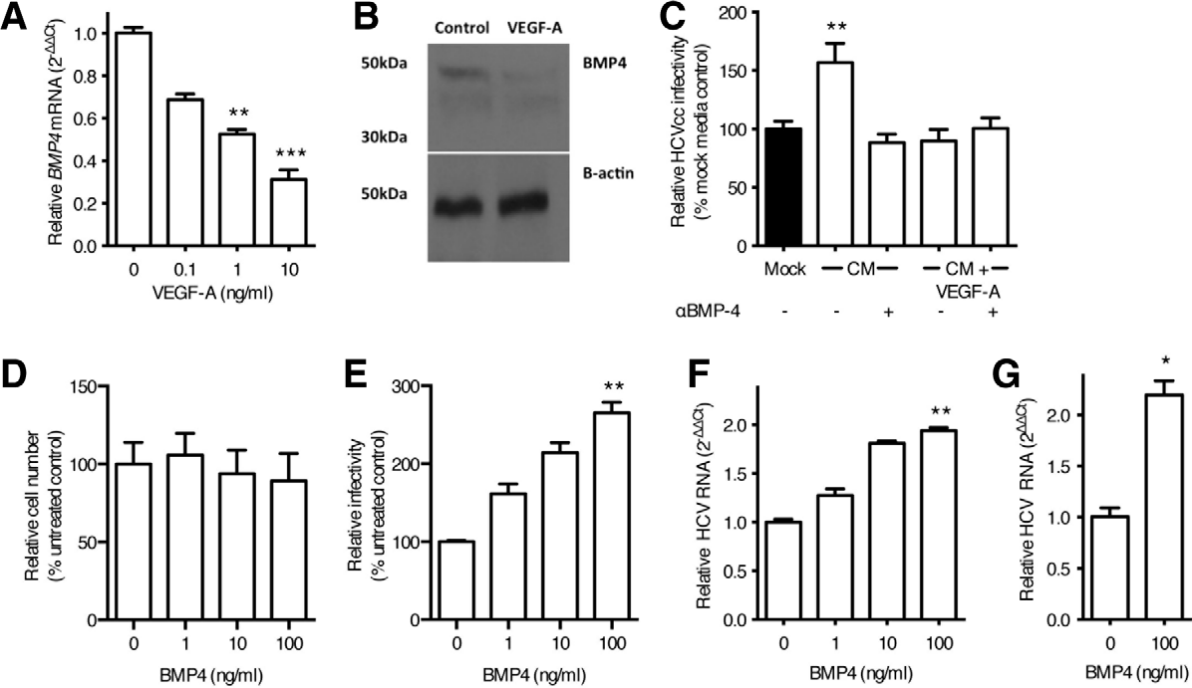
BMP4 is negatively regulated by VEGF and stimulates HCV replication. LSEC were starved of VEGF overnight and stimulated with VEGF-A as indicated for 8 hours and lysed to quantify *BMP4* mRNA (A) or protein (B). Conditioned media (CM) from untreated or VEGF-A treated LSEC were incubated with a neutralizing anti-BMP4 antibody (10 μg/mL) and screened for its effect on Huh-7.5 permissivity (C). Huh-7.5 were treated with recombinant BMP4 for 48 hours and the cells assessed for their proliferative capacity (D) and permissivity to support HCV JFH-1 infection. Infection was enumerated by quantifying NS5A-expressing cells (E) or HCV RNA (F). Primary hepatocytes were treated with BMP4 (100 ng/mL) for 18 hours, inoculated with HCV JFH-1, and infection assessed by measuring HCV RNA (G). Data are mean ±1 SD of n = 4 donor LSEC. Statistical comparisons were made with the Kruskal-Wallis test with Dunn's correction (A,C,E,F), or the Mann-Whitney *U* test (G) and where *P < 0.05, **P < 0.01, and ***P < 0.001 versus mock media or untreated control.

To identify LSEC-expressed proviral factor(s) we used two complementary strategies. First, molecular weight cutoff filters allowed us to show that proviral activity in LSEC CM was >30 kDa and <50 kDa in size (Supporting Fig. 2). Second, messenger RNA (mRNA) microarray identified several transcripts that were regulated by VEGF-A in both LSEC and HUVEC. Differentially regulated transcripts were validated by qRT-PCR (Supporting Fig. 3) and those encoding a predicted soluble or secreted product identified by DAVID analysis.^16^ Four genes were identified that were negatively regulated by VEGF-A in both endothelial cell types: *CCL2, CXCL1, BMP4*, and *CXCL2*. Of these only *BMP4* encodes a protein with a molecular weight compatible with the fractionation studies. LSEC were confirmed to express *BMP4* and expression was down-regulated by VEGF-A (Fig. 3A,B). Furthermore, antibody neutralization of BMP4 in LSEC CM ablated the proviral activity of the CM (Fig. 3C). Importantly, treating Huh-7.5 cells or primary human hepatocytes with recombinant BMP4 resulted in a dose-dependent increase in the number of infected cells (Fig. 3D) and HCV RNA levels (Fig. 3E,F) with minimal effect on cellular proliferation. In summary, these experiments demonstrate that LSEC secrete BMP4 and this promotes hepatocellular permissivity to support HCV replication.

### VEGF-A Regulates BMP4 Expression in a VEGFR-2 and p38 MAPK-Dependent Manner

**Figure 4:**
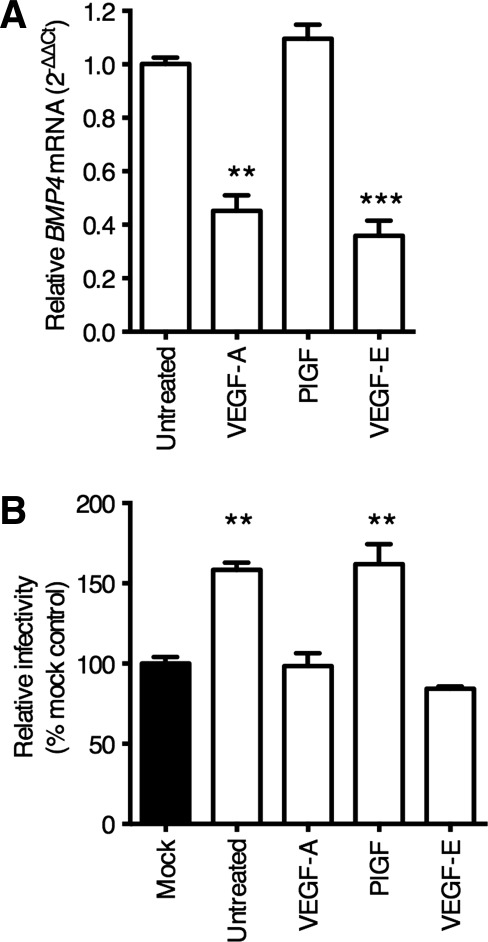
Endothelial BMP4 expression is regulated by way of VEGFR-2. LSEC were starved of VEGF overnight and stimulated with VEGF-A, PlGF, or VEGF-E (all 10 ng/mL) for 8 hours, lysed, and RNA prepared for qRT-PCR analysis of *BMP4* mRNA (A). Conditioned media (CM) from LSEC treated with the above ligands was collected and used to treat Huh-7.5 cells for 18 hours prior to infecting with HCV JFH-1 (B). Data are mean ±1 SD of n = 4 donor LSEC. Statistical comparisons were made with the Kruskal-Wallis test with Dunn's correction as appropriate and where **P < 0.01, and ***P < 0.001, versus untreated control, or mock media as indicated.

VEGF isoforms that bind different receptors enabled us to study the mechanism underlying VEGF-A regulation of BMP4. VEGF-E selectively binds VEGFR-2 and suppresses *BMP4* mRNA to similar levels as VEGF-A, whereas PlGF that targets VEGFR-1 had no effect (Fig. 4A). To confirm a role for VEGFR-2 in LSEC modulation of HCV replication, the endothelial cells were treated with VEGF-A or VEGF-E and the CM screened for its effect on HCV infection of Huh-7.5 cells. Consistent with the model that VEGFR-2 activation negatively regulates BMP4, VEGF-E or VEGF-A ablated the proviral activity of the CM (Fig. 4B). These data show that ligation of endothelial VEGFR-2 limits BMP4 expression and hepatocellular HCV replication.

**Figure 5:**
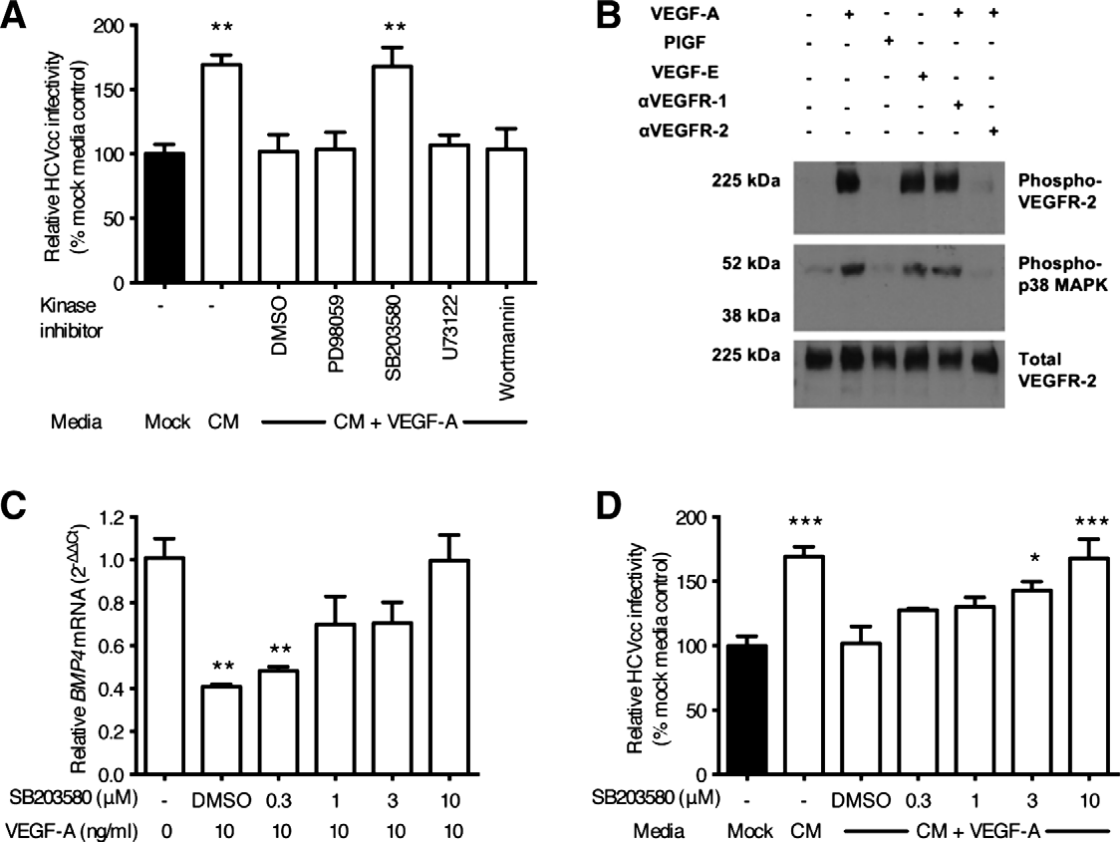
p38 MAPK activation negatively regulates BMP4 expression. LSEC were pretreated with control solvent or kinase inhibitors (all 10 μM) for 1 hour, washed, and incubated with VEGF-A (10 ng/mL) as indicated. Conditioned media (CM) was collected and used to treat Huh-7.5 cells for 18 hours prior to infecting with HCV JFH-1 (A). LSEC were incubated with anti-VEGFR neutralizing antibodies for 1 hour prior to treating with VEGF ligands for 10 minutes before lysing for immunoblotting (B). LSEC were incubated with increasing concentrations of SB203580, or solvent control, before washing and treating with VEGF-A (10 ng/mL) for 8 hours. Cellular *BMP4* levels were measured by qRT-PCR (C) or CM collected and used to treat Huh-7.5 cells for 18 hours prior to infecting with HCV JFH-1 (D). Data are mean ±1 SD of n = 4 donor LSEC. Statistical comparisons were made with the Kruskal-Wallis test with Dunn's correction where *P < 0.05, **P < 0.01, and ***P < 0.001 versus mock media or untreated control as indicated.

To identify VEGF-dependent signals that regulate endothelial *BMP4* expression we screened pharmacological inhibitors of known downstream pathways. Of the inhibitors tested, only p38 MAPK inhibitor SB203580^18^ ablated the effect of VEGF-A stimulation on the proviral activity of LSEC CM (Fig. 5A). VEGF-A activation of LSEC increases the level of phosphorylated p38 MAPK in a VEGFR-2-dependent manner (Fig. 5B), and SB203580 increased LSEC *BMP4* expression following VEGF-A treatment in a dose-dependent manner (Fig. 5C). Finally, treating LSEC with SB203580 prior to VEGF-A stimulation restored HCV infectivity in a dose-dependent manner (Fig. 5D). These experiments demonstrate that VEGF-A inhibits endothelial expression of BMP4 by way of VEGFR-2 mediated p38 MAPK signaling.

### Increased BMP4 Expression in the Inflamed Liver

**Figure 6:**
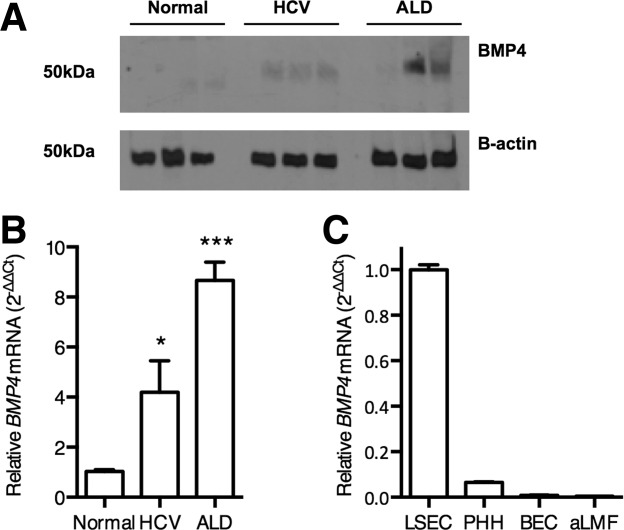
Elevated BMP4 in chronic liver disease. Liver biopsy protein lysates (30 μg) (A) or RNA (B,C) from normal, HCV-infected or alcohol-related liver disease (each n = 6) were immunoblotted for BMP4 (A) and qRT-PCR used to quantify *BMP4* expression (B). Western blot depicts three representative samples and *BMP4* expression is shown relative to normal samples and represents the mean value of all samples. Statistical comparison was made using the Kruskal-Wallis test with Dunn's correction where *P < 0.05, and ***P < 0.001 versus normal liver samples. *BMP4* expression in isolated human liver cell populations was measured by qRT-PCR and expressed relative to LSEC (PHH, primary human hepatocytes; BEC, biliary epithelial cells; aLMF, activated liver myofibroblasts; n = 3 for each cell type) (C).

To determine the effect of liver disease on BMP4 expression we assessed mRNA and protein expression in tissue from age-matched organ donors and patients with HCV infection or alcohol-related liver disease (ALD). We observed negligible BMP4 expression in the normal liver, and a significant increase in both mRNA and protein in the diseased samples (Fig. 6A,B). Attempts to stain BMP4 in liver tissue were unsuccessful; however, qRT-PCR screening a variety of cell types isolated from normal or diseased liver only detected *BMP4* mRNA in LSEC (Fig. 6C).

**Figure 7:**
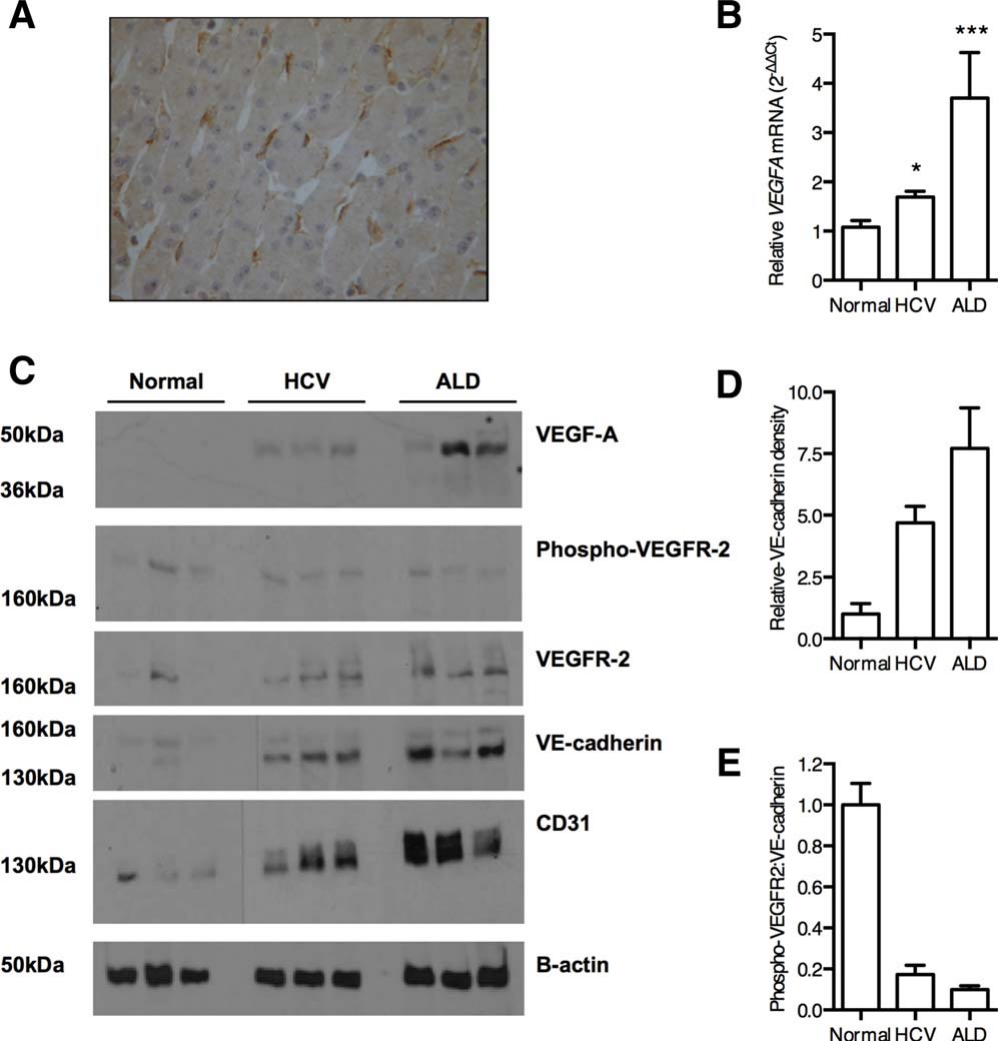
Reduced endothelial VEGFR-2 signaling in chronic liver disease. Representative image of VEGFR-2 expression in normal human liver showing a predominant sinusoidal staining pattern (A). *VEGF-A* expression in normal and diseased liver samples (n = 6) was quantified by qRT-PCR (B). Liver biopsy protein lysates (30 μg) from normal, HCV-infected, or alcohol-related liver disease (each n = 6, representative blots from three donors shown) were probed for proteins of interest as indicated (C). Densitometry was used to quantify VE-cadherin (D) and phosphorylated VEGFR-2 expression, allowing us to determine their relative expression in all samples (E). Statistical comparisons were made with the Kruskal-Wallis test with Dunn's correction where *P < 0.05, and ***P < 0.001 versus normal liver samples.

To confirm our *in vitro* observations that VEGF regulates BMP4 expression in a VEGFR-2-dependent manner, we measured VEGF-A, and basal and phosphorylated VEGFR-2 in liver tissue. Consistent with previous reports we detected a predominantly sinusoidal pattern of VEGFR-2 expression in human liver tissue (Fig. 7A).^6^,^19^ We observed increased VEGF-A and VEGFR-2 levels in diseased liver (Fig. 7A-C), consistent with reports of angiogenesis associated with cirrhosis (reviewed^20^). Importantly, there was a substantial increase in CD31-expressing endothelial cells in diseased liver samples (Supporting Fig. 4). To assess VEGFR-2 activation we quantified phosphorylated protein by immunoblotting and observed variable receptor phosphorylation in normal and diseased tissue (Fig. 7C,D) with no significant differences between these conditions. Given the increased frequency of endothelial cells noted in the diseased liver (Supporting Fig. 4), we probed the western blots for endothelial markers CD31 and VE-cadherin and noted increased expression (Fig. 7C,D), allowing us to normalize phosphorylated VEGFR-2 levels to total endothelial cell mass (Fig. 7E). These studies highlight an absolute increase in endothelial cell numbers with minimal change in the level(s) of activated VEGFR-2, suggesting an overall reduction in VEGFR-2 phosphorylation on a per-cell basis. This reduction in endothelial VEGFR-2 activation associated with increased BMP4 expression and validates our *in vitro* observations.

**Figure 8:**
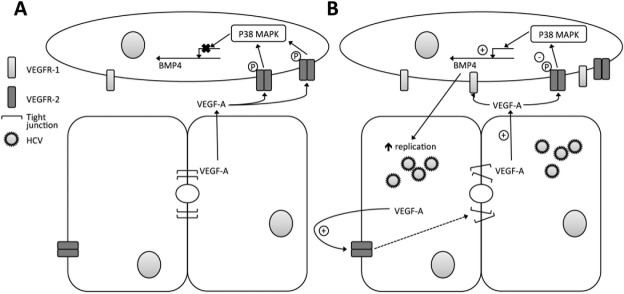
LSEC paracrine signals regulate HCV replication. In the normal liver BMP4 expression is suppressed by way of VEGFR-2/p38 MAPK signaling (A). However, in the HCV-infected or diseased liver, reduced endothelial VEGFR-2 activation permits BMP4 expression that stimulates hepatocellular permissivity to support HCV replication (B).

VEGFR-1 has been reported to regulate VEGFR-2 activity^21^ and we confirmed that *in vitro* stimulation of LSEC with VEGF-A induced *VEGFR-1* expression (Supporting Fig. 5A), suggesting that elevated VEGFR-1 levels observed in the diseased liver may explain the reduction in phosphorylated VEGFR-2. Consistent with this model we observed a significant increase in *VEGFR1* mRNA levels by qRT-PCR in both HCV and ALD tissue (Supporting Fig. 5B). These *ex vivo* data support our *in vitro* findings that endothelial VEGFR-2 activation limits BMP4 expression. In summary, this study highlights a new paracrine pathway for VEGF-A to negatively regulate sinusoidal endothelial expression of BMP4 that can facilitate HCV replication (Fig. 8).

## Discussion

We report that BMP4 promotes HCV replication in hepatocytes and sinusoidal endothelial cells are the major source of BMP4 in the diseased liver. We show that BMP4 is negatively regulated by VEGF-A signaling by way of a VEGFR-2-dependent pathway. We observed increased VEGF-A expression in liver samples from subjects with HCV-associated liver disease and with ALD; however, this was associated with a reduction in per endothelial cell VEGFR-2 activation and increased BMP4 expression, demonstrating the value of studying both ligand and receptor phosphorylation status. The increased levels of BMP4 observed in the diseased liver may represent a physiological response to injury, since BMP4 has been reported to play a role in liver development^22^ and regeneration.^23^ We propose that HCV has evolved to exploit the elevated levels of BMP4 expression in the inflamed liver to promote viral replication.

BMP4 is a member of the transforming growth factor (TGF)-β superfamily. This family of growth factors bind type I and type II serine-threonine kinase receptors and transduce intracellular signals through both Smad and non-Smad-dependent pathways.^24^ There are conflicting reports on the effect of TGF-β on HCV replication^25^,^26^; however, a recent small interfering RNA (siRNA) screen to identify host pathways in the viral lifecycle reported that Smad5 knockdown significantly reduced HCV replication.^27^ In summary, these data provide general support for our observations of a proviral effect of BMP4 on HCV replication.

VEGF-A is the key regulator of endothelial cell phenotype and is critical for liver development and regeneration. Our studies suggest that VEGF-A activation of the sinusoidal endothelium suppresses BMP4 expression in a healthy liver, maintaining an environment that is less favorable for HCV replication. LeCouter et al.^5^ reported that the major effects of VEGF-A in protecting the liver from chemical injury in a murine model were mediated by way of VEGFR-1; in contrast, our studies demonstrate a role for endothelial VEGFR-2-mediated p38 MAPK activation in regulating BMP4 expression. HCV infection has been reported to stimulate VEGF-A expression^8^–^10^ that may explain the elevated levels of VEGFR-1 levels we and others^28^ observed in the infected liver. The function(s) of VEGFR-1 are complex and include acting as a decoy molecule to trap VEGF-A and thereby limit VEGFR-2 activation.^21^ Mahasreshti et al.^29^ reported that increased levels of soluble VEGFR-1 drive liver injury by modulating VEGFR-2 activity. Thus, increased hepatic VEGFR-1 levels may limit VEGFR-2 activation and promote BMP4 expression in the diseased liver.

Our studies uncover a new paracrine network linking endothelial and epithelial cells of the liver. We previously reported that VEGF-A reduced hepatocyte permeability and promoted HCV entry in HepG2 cells,^8^ suggesting that anti-VEGF therapies may limit HCV infection. While this observation appears to contradict the findings presented here, we believe the principal difference between published studies and the current one is explained by the presence of endothelial cells in the experimental systems. We demonstrate that BMP4 stimulates HCV RNA replication and antagonizing VEGF-A in the LSEC-hepatoma coculture promotes viral replication. In contrast, VEGF-A acts directly on polarized hepatocytes to reduce their permeability and promotes virus particle entry. Hence, the relative concentrations of VEGF-A and BMP4 in the liver are likely to impact different steps of the HCV lifecycle. Our observations caution against targeting VEGF-A and highlight the need for models that recapitulate the hepatic microenvironment to screen host targeting adjunctive therapies. It is noteworthy that the current chimeric mouse models that support HCV infection do not recapitulate the normal sinusoidal environment between human hepatocytes and mouse LSEC^30^ and may provide limited observations on the complex biology of the human liver.

BMP4 is implicated in hepatic fibrosis and animal studies demonstrate increased BMP4 expression in activated hepatic stellate cells.^31^ In contrast, we observed low levels of *BMP4* mRNA in activated liver myofibroblasts isolated from diseased liver samples. It is interesting to note that LSEC have been reported to maintain stellate cells in a resting phenotype by way of VEGF stimulated expression of nitric oxide.^32^ Consequently, factors that limit VEGF-A activity are likely to promote fibrogenesis through reduced nitric oxide synthesis and increased BMP4 expression. BMP4 has also been implicated in the development of several cancers including HCC,^33^,^34^ a major cause of mortality in HCV-infected patients. We detected increased *BMP4* and *VEGF* mRNA and protein in liver tissue from patients with alcoholic cirrhosis, suggesting that this pathway is associated with liver injury and may promote HCC-associated pathology by activating hepatocytes and endothelial cells.

In summary, we have discovered a novel interaction between liver endothelial cells and hepatocytes, and in particular a new paracrine network where hepatocellular VEGF-A suppresses endothelial BMP4 expression by activating p38 MAPK by way of VEGFR-2. The observation that BMP4 promotes HCV replication suggests a new therapeutic target for treating chronic hepatitis C. Furthermore, reports showing a role for BMP4 in liver development and in the response to injury suggests that targeting this pathway may have profound effects on pathophysiologically relevant processes in liver disease and carcinogenesis.

## Abbreviations

ALD: alcoholic liver disease
BMP4: bone morphogenetic protein 4
CM: conditioned media
HBV: hepatitis B virus
HCC: hepatocellular carcinoma
HCV: hepatitis C virus
HUVEC: human umbilical vein endothelial cell
LSEC: liver sinusoidal endothelial cell
TGF-β: transforming growth factor β
VEGF: vascular endothelial growth factor
VEGFR: vascular endothelial growth factor receptor
