# Supplementary material for: Paracrine Signals From Liver Sinusoidal Endothelium Regulate Hepatitis C Virus Replication
Source: Hepatology. 2013 Dec 18;59(2):375–84. doi: 10.1002/hep.26571 (PMC3992845; doi:10.1002/hep.26571)
Supplement: Supplementary file 1 [file hep0059-0375-sd1.docx]

**Paracrine signals from liver sinusoidal endothelium regulate HCV replication**

Rowe et al.

**Supporting information**

**Supporting figures**

**Supporting Fig. 1. Response of independent donor LSEC to VEGF-A stimulation.** Conditioned media (CM) were collected from LSEC seeded at 4x10^4^/cm^2^ for 24 hours in the presence or absence of VEGF-A (10 ng/ml), diluted 1:2 with fresh media and used to treat Huh-7.5 for 18 hours prior to infecting with HCV JFH-1. CM was replenished following infection and the frequency of NS5A expressing cells quantified after 72 hours.

**Supporting Fig. 2. Fractionation of LSEC conditioned media**. Conditioned media (CM) was collected from LSEC cultured in the presence (**A**) or absence of VEGF (**B**) for 18 hours. In each case the media were fractionated using molecular weight cut-off filters and used to treat Huh-7.5 cells prior to infecting with HCV. Infectivity data is presented relative to Huh-7.5 cells treated with non-conditioned endothelia or mock media. Statistical comparisons were made using the Kruskall-Wallis test with Dunn’s correction where * *P*<0.05, and ** *P*<0.01, vs. mock endothelial cell media.

**Supporting Fig. 3. Validation of relevant transcripts identified in LSEC microarray**. LSEC from 3 independent donors were treated with VEGF-A (10ng/ml) for 18 hours, cells lysed and RNA prepared for qRT-PCR analysis. **A**. Predicted upregulated genes: *PLAT*, *VASH*, and *VEGFR-1*. **B**. Predicted downregulated transcripts: *CXCL1*, and *CCL2*. Gene expression is shown relative to untreated control cells and represents the mean value of all the samples.


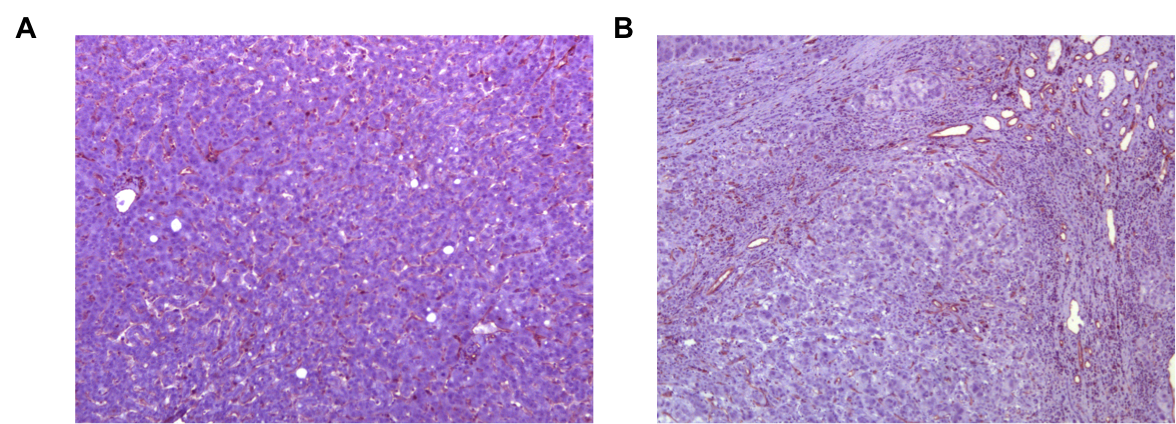


**Supporting Fig. 4. CD31 expression in liver.** Representative images showing CD31 expression in normal liver (**A**) and HCV infected liver tissue (**B**). Magnification x40..

**Supporting Fig. 5. VEGFR-1 expression is regulated by VEGF and is increased in diseased liver.** LSEC were treated with increasing concentrations of VEGF-A as indicated. *VEGFR-1* expression was determined by qRT-PCR (**A**). *VEGFR-1* expression in total liver RNA from 6 normal, HCV and ALD biopsy samples (**B**). Data are presented relative to expression of *GAP*DH and represent the mean value of all samples. Statistical comparisons were made with the Mann-Witney U test, or the Kruskall-Wallis test with Dunn’s correction as appropriate and where * *P*<0.05, and ** *P*<0.01, vs. untreated control or normal liver as indicated.
